# Should Patients with Traumatic Brain Injury with Significant Contusions be Treated with Different Neurointensive Care Targets?

**DOI:** 10.1007/s12028-024-01954-y

**Published:** 2024-03-20

**Authors:** Teodor Svedung Wettervik, Anders Hånell, Anders Lewén, Per Enblad

**Affiliations:** Department of Medical Sciences, Section of Neurosurgery, Uppsala University, 751 85 Uppsala, Sweden

**Keywords:** Cerebral perfusion pressure, Contusions, Neurointensive care, Outcome, Pressure reactivity, Traumatic brain injury

## Abstract

**Background:**

Patients with traumatic brain injury (TBI) with large contusions make up a specific TBI subtype. Because of the risk of brain edema worsening, elevated cerebral perfusion pressure (CPP) may be particularly dangerous. The pressure reactivity index (PRx) and optimal cerebral perfusion pressure (CPPopt) are new promising perfusion targets based on cerebral autoregulation, but they reflect the global brain state and may be less valid in patients with predominant focal lesions. In this study, we aimed to investigate if patients with TBI with significant contusions exhibited a different association between PRx, CPP, and CPPopt in relation to functional outcome compared to those with small/no contusions.

**Methods:**

This observational study included 385 patients with moderate to severe TBI treated at a neurointensive care unit in Uppsala, Sweden. The patients were classified into two groups: (1) significant contusions (> 10 mL) and (2) small/no contusions (but with extra-axial or diffuse injuries). The percentage of good monitoring time (%GMT) with intracranial pressure > 20 mm Hg; PRx > 0.30; CPP < 60 mm Hg, within 60–70 mm Hg, or > 70 mm Hg; and ΔCPPopt less than − 5 mm Hg, ± 5 mm Hg, or > 5 mm Hg was calculated. Outcome (Glasgow Outcome Scale-Extended) was assessed after 6 months.

**Results:**

Among the 120 (31%) patients with significant contusions, a lower %GMT with CPP between 60 and 70 mm Hg was independently associated with unfavorable outcome. The %GMTs with PRx and ΔCPPopt ± 5 mm Hg were not independently associated with outcome. Among the 265 (69%) patients with small/no contusions, a higher %GMT of PRx > 0.30 and a lower %GMT of ΔCPPopt ± 5 mm Hg were independently associated with unfavorable outcome.

**Conclusions:**

In patients with TBI with significant contusions, CPP within 60–70 mm Hg may improve outcome. PRx and CPPopt, which reflect global cerebral pressure autoregulation, may be useful in patients with TBI without significant focal brain lesions but seem less valid for those with large contusions. However, this was an observational, hypothesis-generating study; our findings need to be validated in prospective studies before translating them into clinical practice.

**Supplementary Information:**

The online version contains supplementary material available at 10.1007/s12028-024-01954-y.

## Introduction

In moderate to severe traumatic brain injury (TBI), management is guided toward emergency evacuation of traumatic lesions with mass effect [1] and neurointensive care to reduce the development of secondary brain injury [2–4]. Traditional neurointensive care aims at keeping systemic and cerebral physiological variables within fixed targets, e.g., intracranial pressure (ICP) below 20–22 mm Hg and cerebral perfusion pressure (CPP) between 60 and 70 mm Hg [2, 3, 5]. Although this approach has led to tremendous improvements in survival and functional recovery [3, 6], fixed treatment targets for all patients may be too crude, considering the heterogeneous nature of the disease [7]. Thus, treatments based on patient-specific demographics, advanced software analysis of neuromonitoring data, and classifications of intracranial injury patterns could be a way toward precision medicine in neurointensive care [2]. One suggested approach in this direction is to adapt CPP management based on the cerebral pressure autoregulatory status in each patient [8–12]. The cerebral autoregulatory status can be continuously monitored as the correlation coefficient between ICP and arterial blood pressure (ABP) [12–15], such as the pressure reactivity index (PRx) [14]. The cerebral autoregulatory status differs between patients and changes over time after TBI [12, 16]. Some studies have found that the optimal absolute CPP interval may differ depending on the cerebral autoregulatory status [13, 17]. For example, patients with pressure passive cerebral vessels and impaired cerebral autoregulation may benefit from slightly lower CPP to avoid hyperemia and worsening of brain edema [13]. In addition, PRx often varies with CPP in a U-shaped way, corresponding to the Lassen curve, and the CPP with the lowest PRx (best cerebral pressure autoregulation) has been suggested to be optimal (CPPopt) [10, 11, 18, 19]. Deviations from CPPopt (ΔCPPopt = actual CPP − CPPopt) has been associated with worse brain oxygenation [20, 21], disturbed energy metabolism [22], and unfavorable outcome [10–12] in observational studies, and CPPopt was shown to be a safe and feasible target in a prospective trial [9].

Furthermore, patients with TBI exhibit a wide array of one or more traumatic intracranial lesion features [1, 7, 23]. This may be of relevance for treatment in neurointensive care because the optimal physiological targets may differ among patients depending on the intracranial injury pattern. In an earlier pilot study, we found that patients with a focal brain injury benefited from lower CPP levels, whereas the opposite was found for those with a diffuse injury if the cerebral pressure autoregulation was disrupted [24]. However, these analyses were limited by a small patient cohort (*n* = 107), and a crude classification into diffuse vs. focal injury according to the Marshall grade [25] was used. More studies are warranted concerning whether CPP management should be individualized according to the type of brain injury and the significance of cerebral autoregulatory status in different injury types. In particular, high CPP is likely more dangerous in patients with cerebral contusions because they suffer from significant blood–brain barrier disruption [26] and more easily develop brain edema [27]. At the same time, it is possible that the global variables of cerebral pressure autoregulation (PRx and CPPopt) may be insensitive to the pericontusional autoregulatory status and blood flow disturbances that may occur in the presence of a significant focal cerebral contusion [19].

To explore further ways of precision approaches of neurointensive care, we investigated the role of absolute CPP, PRx, and ΔCPPopt in relation to functional outcome for patients with TBI with significant contusions in relation to those with only small or no contusions. We hypothesized that high CPP would have a stronger association with unfavorable outcome in patients with significant contusions and that the global cerebral autoregulatory variables, PRx and CPPopt, would only be weakly associated with outcome in this TBI subtype because of the focal nature of the injury.

## Methods

### Patients and Study Design

This was an observational study, conducted at the Department of Neurosurgery, Uppsala University Hospital, Uppsala, Sweden. Of 490 patients with TBI who were older than 15 years, received ICP monitoring, had at least two computed tomography (CT) scans the first 48 h, and were treated in our neurointensive care unit between 2008 and 2018, 105 were excluded (missing outcome data = 42, less than 12 h of ICP data the first 7 days = 63). Thus, the final study population included 385 patients.

### Neurointensive Care Management Protocol

The patients were treated according to our standardized ICP-oriented and CPP-oriented TBI management protocol, which has been described in detail in previous studies [3, 28]. Treatment goals were as follows: ICP ≤ 20 mm Hg, CPP ≥ 60 mm Hg, systolic blood pressure ≥ 100 mm Hg, partial pressure of oxygen ≥ 12 kPa, arterial glucose level of 5–10 mM, hemoglobin level ≥ 10 g/dL, electrolytes within normal ranges, normovolemia, and body temperature < 38 °C.

Unconscious (Glasgow Coma Scale Motor score (GCS M 1–5)) patients were intubated, mechanically ventilated, and given propofol and morphine for sedation and analgesia, respectively. ICP was monitored with an external ventricular device (EVD; HanniSet, Xtrans; Smiths Medical GmbH, Glasbrunn, Germany) or an intraparenchymal sensor device (Codman ICP Micro-Sensor; Codman & Shurtleff, Raynham, MA) in unconscious patients. Surgical evacuation was performed in patients with significant intracranial mass lesions. The basic management included head elevation to 30° and mild hyperventilation (partial pressure of carbon dioxide 4.0–4.5 kPa) if ICP was increased. Neurological wake-up tests were done three times per day but not if ICP remained elevated. If ICP was still increased in the absence of mass lesions, an EVD was used to drain cerebrospinal fluid. If ICP was still refractory elevated, stress was treated with increased sedation, β_1_-antagonists, and α_2_-agonists. Last-tier treatments to control ICP included thiopental infusion and/or decompressive craniectomy (DC).

### Functional Outcome

Functional outcome was evaluated according to the Glasgow Outcome Scale-Extended (GOS-E) 6 months post injury. The scale has eight categories and ranges from death (1) to upper good recovery (8) [29, 30]. The assessments were conducted by specially trained staff using structured telephone interviews with the patients if they had recovered sufficiently and otherwise with their closest relative. Favorable and unfavorable outcomes were classified as GOS-E scores 5–8 and 1–4, respectively.

### Radiological Analysis

Volumetric (mL) assessments of contusions were performed based on the first two CT scans in the Brainlab software by one of the authors (TSW). To also assess the extra-axial hemorrhage burden, similar volumetric assessments were conducted for epidural hematomas (EDHs), acute subdural hematomas (aSDHs), and intraventricular hemorrhage (IVH). Only the sum of each hematoma type was reported (e.g., a reported contusion volume of 10 mL could reflect two contusions of 5 mL each). Presence/absence of traumatic subarachnoid hemorrhage (tSAH) on any of these two scans was also assessed; however, it could not be properly quantified volumetrically. Furthermore, the midline shift and the extent of compression of the basal cisterns (open, compressed, or obliterated) were evaluated. The highest value of the bleeding volume, midline shift, and basal cistern compression on any of the two CT scans was used in the statistical analyses. The Marshall classification was also assessed based on the first CT scan by the same author (TSW) [25]. We used 10-mL contusion volume as the cutoff to dichotomize patients into (1) significant contusion and (2) small/no contusion (but potentially extra-axial or diffuse injuries). We chose this cutoff a priori because it included contusions that were large enough to be significant, although they did not necessarily require surgical evacuation in all cases (e.g., surgery was usually only done for contusions sized 25 mL and above) [25]. The cutoff at 10 mL was similar to that in the Surgical Trial In Traumatic intraCerebral Haemorrhage (STITCH) trial [31].

### Data Acquisition and Analysis

The physiological variables (ABP and ICP) were recorded at 100 Hz using the Odin software [13]. ABP was measured in the radial artery at heart level. ICP was monitored with either an EVD with the transducer at the level of foramen of Monro or an intraparenchymal probe. If a patient had both an intraparenchymal monitor and an EVD, only the ICP from the EVD was registered and analyzed. PRx was calculated as the 5-min correlation of 10-s averages of ICP and mean ABP [12, 14]. CPPopt was continuously calculated as the CPP with the lowest PRx the last 4 h [10–12]. The physiological variables were down-sampled and analyzed as minute-by-minute values. PRx and CPPopt were calculated in retrospect and were not available at the bedside. The physiological variables were analyzed during the first 7 days after injury.

Good monitoring time (GMT) was defined as the total monitoring time (in minutes) subtracted by the time when the data acquisition was interrupted (e.g., when the patients left neurointensive care for surgery) and after removal of artifactual values. The %GMTs with ICP > 20 mm Hg, PRx > 0.30, CPP < 60 mm Hg, CPP within 60–70 mm Hg, CPP > 70 mm Hg, ΔCPPopt less than − 5 mm Hg, ΔCPPopt ± 5 mm Hg, and ΔCPPopt > 5 mm Hg were analyzed during the first 7 days post injury. The ICP threshold was chosen according to our management protocol [3]. The CPP thresholds were defined according to the Brain Trauma Foundation guidelines [5]. We chose PRx > 0.30 as a threshold because PRx between 0.20 and 0.40 has been associated with unfavorable outcome [32] and has also been estimated as the lower limit of autoregulation [8]. The ΔCPPopt threshold at 5 mm Hg was chosen in accordance with the CPPopt Guided Therapy: Assessment of Target Effectiveness (COGiTATE) trial [9].

### Visualization of Combined Insults

To study the combined role of the absolute PRx/CPP thresholds a two-dimensional plot was conducted, which illustrated the correlation between %GMT of various PRx/CPP combinations and GOS-E. This method has been developed by our group and has been described in detail in a previous study [17]. These plots were created for the significant contusion and the small/no contusion groups. The %GMT during the first 7 days after injury for combinations of PRx (range − 1 to + 1 with a 0.05 resolution) and CPP values (range 40–100 mm Hg, with a 2-mm Hg resolution), yielding a grid of 1,200 cells (40 × 30), was calculated for all patients in each cohort and analyzed in relation to GOS-E with the Spearman test. To reduce high-frequency noise, each pixel was divided into 3 by 3 smaller pixels, followed by a Gaussian smoothing (standard deviation = 2). The final values for each pixel were translated into the jet color range (red to blue) with red/blue color indicating a negative/positive association with GOS-E. The jet color scale was limited to a ± 0.30 correlation coefficient range because of the moderate correlation strength. Pixels with fewer than five patients with at least 5 min of monitoring time were colored as white. Furthermore, a density plot was conducted to visualize the frequency of the %GMT for certain combinations of PRx and CPP. The resulting numbers were divided by the highest count within the grid to yield density values ranging from 0 to 1 for each cell in the grid. Gaussian smoothing was also applied here, and the final values were then transformed to colors using the jet color scale and plotted. Furthermore, similar plots were done with ∆CPPopt, instead of CPP, in combination with PRx in relation to GOS-E.

### Statistical Analysis

Nominal variables were presented as numbers (proportions), and ordinal/continuous variables were presented as medians (interquartile range [IQR]). Differences in demographics, admission variables, treatments, outcome, imaging, and cerebral physiology between those with significant contusions and those with small/no contusions were evaluated with the Mann–Whitney *U*-test or the χ^2^ test, depending on the type of data. The association of the %GMTs within/outside the thresholds of ICP, PRx, CPP, and ΔCPPopt with GOS-E was assessed with the Spearman test for the significant contusions and the small/no contusions groups, separately. Multiple logistic regressions with unfavorable outcome (GOS-E scores 1–4) as the dependent variable and age, GCS M, pupillary status, and the %GMTs of ICP > 20 mm Hg, PRx > 0.30, and CPP within 60 to 70 mm Hg as independent variables were conducted for the significant contusions and the small/no contusion groups, separately. The independent variables were similar to those in the International Mission for Prognosis and Clinical Trials in Traumatic Brain Injury (IMPACT) core model (age, GCS M, and pupillary status) [33], in addition to being within/outside the explored ICP, PRx, and CPP thresholds. We chose CPP within 60–70 mm Hg because this interval was more strongly associated with GOS-E in univariate analysis than values below or above this interval. In similar regressions, the %GMT of CPP within 60–70 mm Hg was replaced by the autoregulatory target ΔCPPopt ± 5 mm Hg. We chose ΔCPPopt ± 5 mm Hg because this interval was more strongly associated with GOS-E in univariate analysis than values below or above this interval. Missing data were rare (CPPopt could not be calculated in six patients with otherwise complete data), and these patients were excluded from the relevant analyses, i.e., no imputation was done. A *p* value < 0.05 was considered statistically significant. We abstained from adjustment for multiple comparisons because this was an exploratory study. The statistical analyses were conducted in RStudio software (version 2022.12.0) [34].

### Ethics

All procedures performed in the studies involving humans were in accordance with the ethical standards of the national research committee and with the 1964 Helsinki Declaration and its later amendments. The study was approved by Uppsala University Regional Ethical Board (Dnr: 2022–06526-02). Written informed consent was obtained during neurointensive care or follow-up by most patients or their relatives but was waived if the patient/relatives could no longer be reached.

## Results

### Demographics, Admission Variables, Clinical Course, Treatments, and Outcome

In the entire cohort of 385 patients, 120 (31%) had a significant contusion and the remaining 265 (69%) patients had small/no contusions (Table 1). The median age was 52 (IQR 31–65) years, and 78% of patients were male. At admission, the median GCS M score was 5 (IQR 4–5), and 20% of patients exhibited unreactive pupil(s). Around 10% of patients were treated with last-tier ICP treatments, including thiopental infusion and DC. At 6 months post-TBI, 53% of patients had recovered favorably (GOS-E scores 5–8) and 16% were deceased. Those with significant contusions were slightly older, presented with a higher GCS M score, were more often underwent a craniotomy for hematoma evacuation, and showed worse functional recovery with a lower GOS-E score at follow-up.

**Table 1:** Demographics, admission variables, treatments, and functional outcome

Variables	All	Significant contusions	Small/no contusions	*p*
Patients, *n* (%)	385 (100%)	120 (31%)	265 (69%)	NA
Age (years), median (IQR)	52 (31–65)	59 (46–68)	48 (26–63)	< 0.001*
Sex (male/female), *n* (%)	300/85 (78%/22%)	97/23 (81%/21%)	203/62 (77%/23%)	0.35
GCS M at admission, median (IQR)	5 (4–5)	5 (5–6)	5 (4–5)	0.02*
Unreactive pupil(s), *n* (%)	76 (20%)	20 (17%)	56 (21%)	0.31
Marshall grade (diffuse injury I-IV/focal injury), *n* (%)	253/132 (66%/34%)	68/52 (57%/43%)	185/80 (70%/30%)	0.01*
ICP monitor (intraparenchymal/EVD/both), *n* (%)	218/77/90 (57%/20%/23%)	60/25/35 (50%/21%/29%)	158/52/55 (60%/20%/21%)	0.14
Hematoma evacuation (yes), *n* (%)	179 (46%)	75 (63%)	104 (39%)	< 0.001*
Thiopental (yes), *n* (%)	49 (13%)	18 (15%)	31 (12%)	0.37
DC (yes), *n* (%)	43 (11%)	19 (16%)	24 (9%)	0.051
GOS-E, median (IQR)	5 (3–7)	4 (3–6)	5 (3–7)	0.01*
Favorable/unfavorable outcome, *n* (%)	203/182 (53%/47%)	52 (43%)	151 (57%)	0.01*
Mortality, *n* (%)	63 (16%)	27 (23%)	36 (14%)	0.03*

Statistical comparisons between the significant contusions and small/no contusions groups were conducted with the Mann–Whitney *U*-test

DC, decompressive craniectomy, EVD, external ventricular drainage, GCS M, Glasgow Comas Scale motor score, GOS-E, Glasgow Outcome Scale-Extended, ICP, intracranial pressure, IQR, interquartile range, NA, not applicable

^*^Statistical significance

### Intracranial Lesion Features

In the entire cohort, 9% had an EDH, 44% had an aSDH, 72% had tSAH, 24% had IVH, and 71% had contusions (Table 2). The median midline shift was 0 (IQR 0–7) mm, and the basal cisterns were compressed or obliterated in 18%. Those in the cohort with significant contusions had tSAH more often and greater midline shift but less often had an EDH than those with small/no contusions (Table 2). The median EDH volume was smaller in the entire significant contusions group compared to the small/no contusions group, but there was no difference in aSDH or IVH volume between the groups (Table 2). Approximately half (*n* = 179) of the patients were operated on for hematoma evacuation (see Supplementary Table 1 for details). All contusion evacuations occurred in the cohort with significant contusions.

**Table 2:** Traumatic intracranial lesion features

Variables	All	Significant contusions	Small/no contusions	*p*
Intracranial hemorrhage				
EDH (yes), n (%)	34 (9%)	5 (4%)	29 (11%)	0.03*
EDH volume (mL), median (IQR)^a^	0 (0–0)	0 (0–0)/53 (18–70)	0 (0–0)/21 (11–65)	0.03*
aSDH (yes), n (%)	171 (44%)	61 (51%)	110 (42%)	0.09
aSDH volume (mL), median (IQR)^a^	0 (0–16)	1 (0–11)/10 (5–27)	0 (0–23)/47 (9–103)	0.71
tSAH (yes), n (%)	279 (72%)	116 (97%)	163 (62%)	< 0.001*
IVH (yes), n (%)	93 (24%)	33 (28%)	60 (23%)	0.30
IVH volume (mL), median (IQR)^a^	0 (0–0)	0 (0–0.2)/0.9 (0.3–3.4)	0 (0–0)/0.5 (0.2–1.5)	0.21
Contusion (yes), n (%)	272 (71%)	120 (100%)	152 (57%)	< 0.001*
Contusion volume (mL), median (IQR)^a^	2 (0–16)	29 (17–68)/29 (17–68)	0.3 (0–3)/2 (0.6–5)	< 0.001*
Mass effect				
Midline shift (mm), median (IQR)	0 (0–7)	4 (0–8)	0 (0–7)	< 0.001*
Basal cisterns (open/compressed/obliterated), n (%)	315/50/20 (82%/13%/5%)	94/21/5 (78%/18%/4%)	221/29/15 (83%/11%/6%)	0.19

Statistical comparisons between the significant contusions and small/no contusions groups were conducted with the Mann–Whitney *U*-test or the χ^2^ test, depending on the type of data. There were 120 patients in the significant contusions cohort and 265 patients in the small/no contusions cohort

aSDH, acute subdural hematoma, EDH, epidural hematoma, IQR, interquartile range, IVH, intraventricular hemorrhage, tSAH, traumatic subarachnoid hemorrhage

^*^Statistical significance

^a^The median (IQR) volume of each intracranial hematoma subtype is described first in the entire cohort (including those 0 mL) and second (after “/”) only in the subgroup with that type of hematoma (excluding those with 0 mL). The significance tests were based on the entire cohort (including those with 0 mL). For that reason, for the EDH subtype, the volume was statistically higher in the small/no contusions group because more patients exhibited this lesion type, although the volume was slightly larger in the small subgroup of patients who had an EDH in the significant contusions group

### Cerebral Physiology During the First 7 Days Post Injury

In the entire cohort (Table 3), the median %GMT with ICP > 20 mm Hg was 3 (IQR 1–10), the median %GMT with PRx > 0.30 was 25 (IQR 18–29), the median %GMT with CPP within 60 to 70 mm Hg was 22 (IQR 11–33), and the median %GMT with ∆CPPopt ± 5 mm Hg was 27 (IQR 23–33). Those with significant contusions exhibited a higher %GMT of ICP > 20 mm Hg than those with small/no contusions, but there was otherwise no difference in these physiological variables between the groups. During the first week, the median number of days with ICP monitoring was 5 (IQR 4–6).

**Table 3:** The burden of cerebral physiological insults during the first 7 days post injury

	All	Significant contusions	Small/no contusions	*p*
ICP				
ICP > 20 mm Hg (%GMT), median (IQR)	3 (1–10)	5 (1–14)	3 (1–10)	0.004*
Pressure autoregulatory insults				
PRx > 0.30 (%GMT), median (IQR)	25 (18–29)	27 (20–36)	23 (17–33)	0.06
Absolute CPP				
CPP < 60 mm Hg (%GMT), median (IQR)	4 (1–9)	4 (1–9)	4 (1–9)	0.94
CPP 60 to 70 mm Hg (%GMT), median (IQR)	22 (11–33)	22 (10–31)	23 (11–35)	0.28
CPP > 70 mm Hg (%GMT), median (IQR)	67 (50–84)	66 (54–86)	67 (48–84)	0.40
CPPopt				
∆CPPopt < − 5 mm Hg (%GMT), median (IQR)	33 (24–43)	33 (24–46)	33 (24–42)	0.56
∆CPPopt ± 5 mm Hg (%GMT), median (IQR)	27 (23–33)	26 (23–32)	28 (23–33)	0.37
∆CPPopt > 5 mm Hg (%GMT), median (IQR)	31 (22–41)	31 (22–42)	32 (22–41)	0.96

Statistical comparisons between the significant contusions and small/no contusions groups were conducted with the Mann–Whitney *U*-test, depending on the type of data. There were 120 patients in the significant contusions cohort and 265 patients in the small/no contusions cohort

CPP, cerebral perfusion pressure, CPPopt, optimal CPP, GMT, good monitoring time, ICP, intracranial pressure, IQR, interquartile range, PRx, pressure reactivity index

^*^Statistical significance

### Relation Between Cerebral Physiological Variables and Outcome in Patients with Significant Contusions and Small/no Contusions

**Fig. 1:**
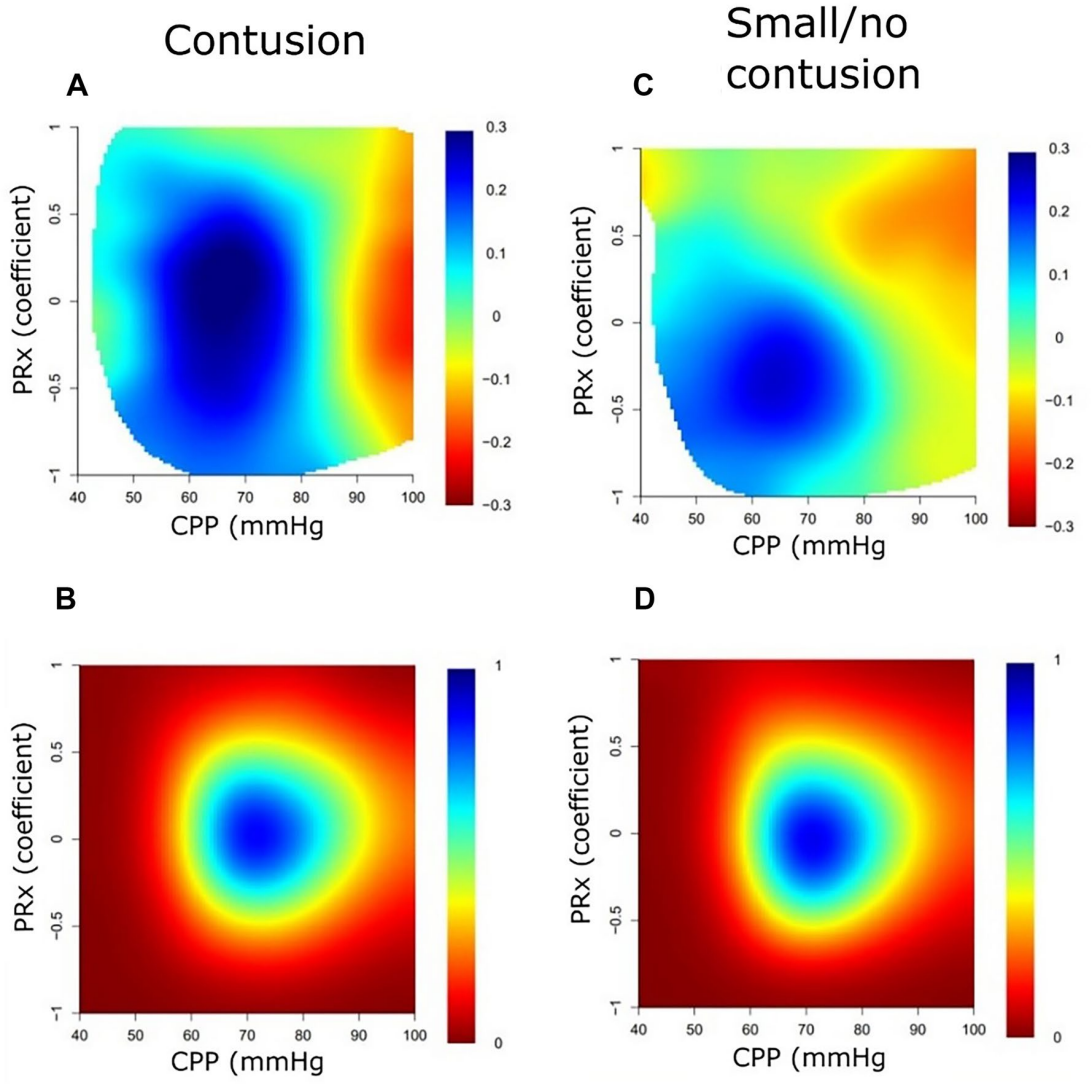
Combined insults of PRx together with absolute CPP the first 7 days after injury: relation to GOS-E and data density. **a**, The %GMT for the concurrent combination of PRx and CPP during the first 7 days after injury was calculated and correlated with GOS-E in the significant contusion cohort. The jet color range denotes the value of the correlation coefficients, where blue color indicates a correlation between a higher %GMT and more favorable outcome (higher GOS-E) and red color indicates a correlation between a higher %GMT and more unfavorable outcome (lower GOS-E). The jet color scale was limited to ± 0.30 correlation coefficient range because of the moderate correlation strength. Pixels with fewer than five patients with at least 5 min of monitoring time were colored as white. **b**, A density plot was conducted to visualize the frequency of the %GMT for certain combinations of PRx and CPP. The resulting numbers were divided by the highest count within the grid to yield density values ranging from 0 to 1 for each cell in the grid. **c** and **d**, Similar %GMT (**c**) and density (**d**) plots were conducted for the combination of PRx and CPP in the small/no contusion cohort. CPP cerebral perfusion pressure, GMT good monitoring time, GOS-E Glasgow Outcome Scale-Extended, PRx pressure reactivity index (Color figure online)

**Fig. 2:**
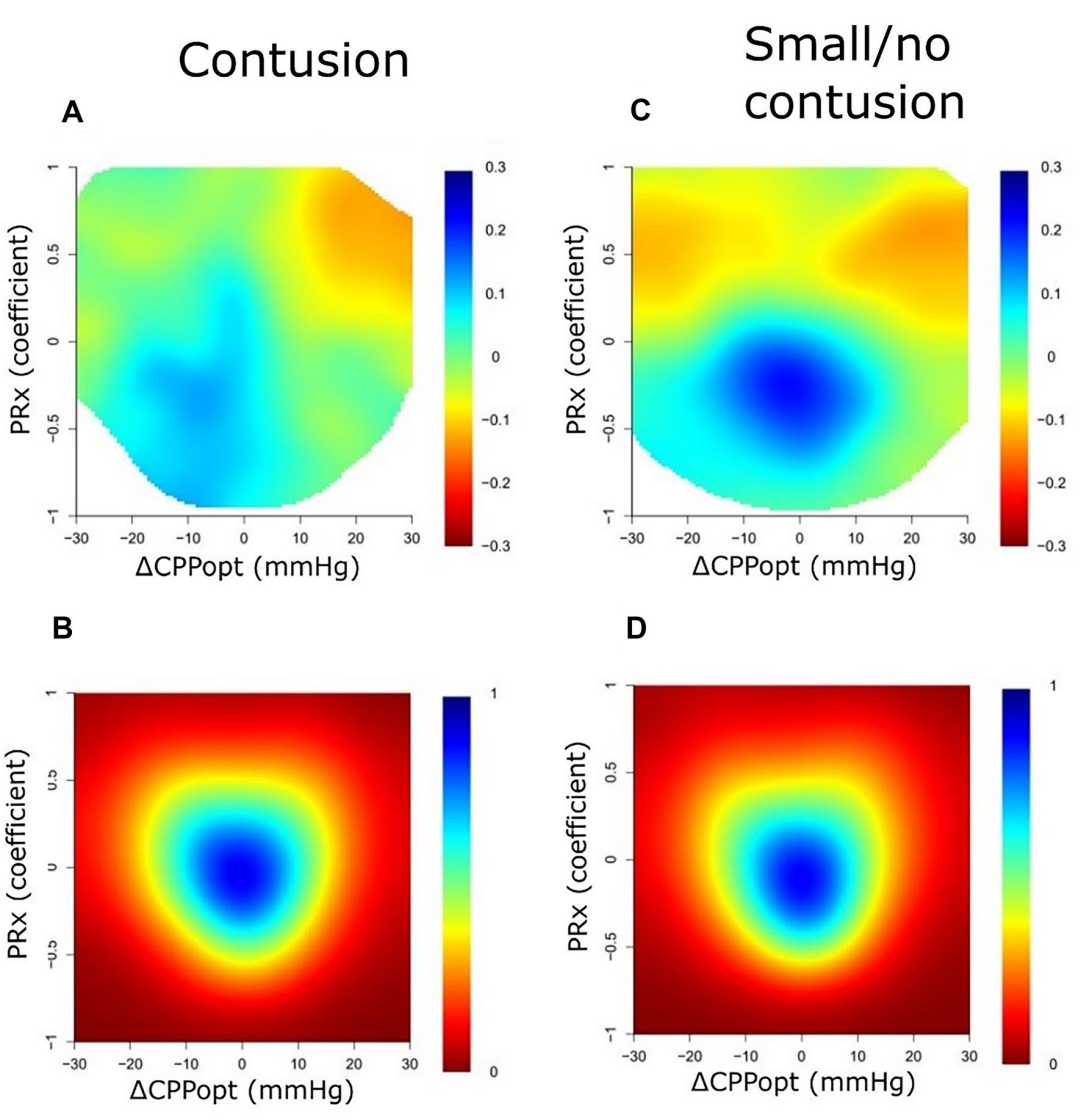
Combined insults of PRx together with ΔCPPopt the first 7 days after injury: relation to GOS-E and data density.** a**, The %GMT for the concurrent combination of PRx and ΔCPPopt during the first 7 days after injury was calculated and correlated with GOS-E in the significant contusion cohort. The jet color range denotes the value of the correlation coefficients, where blue color indicates a correlation between a higher %GMT and more favorable outcome (higher GOS-E) and red color indicates a correlation between a higher %GMT and more unfavorable outcome (lower GOS-E). The jet color scale was limited to ± 0.30 correlation coefficient range because of the moderate correlation strength. Pixels with fewer than five patients with at least 5 min of monitoring time were colored as white. **b**, A density plot was conducted to visualize the frequency of the %GMT for certain combinations of PRx and ΔCPPopt. The resulting numbers were divided by the highest count within the grid to yield density values ranging from 0 to 1 for each cell in the grid. **c** and **d**, Similar %GMT (**c**) and density (**d**) plots were conducted for the combination of PRx and ΔCPPopt in the small/no contusion cohort. CPP cerebral perfusion pressure, CPPopt optimal CPP, GMT good monitoring time, GOS-E Glasgow Outcome Scale-Extended, PRx pressure reactivity index (Color figure online)

In the cohort with significant contusions (Table 4), the %GMTs of ICP > 20 mm Hg and PRx > 0.30 were not associated with GOS-E, whereas CPP < 60 mm Hg, CPP within 60–70 mm Hg, and higher ∆CPPopt ± 5 mm Hg were associated with higher GOS-E scores, and CPP > 70 mm Hg was associated with lower GOS-E scores. Excluding those patients who had undergone DC from the analyses between PRx/∆CPPopt and GOS-E did not alter the associations. In a heatmap with the %GMT of PRx/CPP combinations in relation to GOS-E (Fig. 1a), there was a transition toward unfavorable outcome when CPP exceeded 80 mm Hg for the significant contusions group. The association between CPP and GOS-E did not appear to change depending on the concurrent PRx value (Fig. 1a). In similar analyses of PRx/ΔCPPopt combinations in relation to GOS-E (Fig. 2a), there was a slight transition toward unfavorable outcome for ΔCPPopt > 10 mm Hg when PRx was above approximately 0.25.

**Table 4:** Cerebral physiological insults the first 7 days post injury in relation to GOS-E: a Spearman analysis

Variables	Significant contusions	Small/no contusion
ICP		
ICP > 20 mm Hg (%GMT)	0.08	− 0.05
Pressure autoregulation		
PRx > 0.30 (%GMT)	− 0.17	− 0.32^a^
Absolute CPP		
CPP < 60 mm Hg (%GMT)	0.21^b^	0.04
CPP 60 to 70 mm Hg (%GMT)	0.32^a^	0.20^c^
CPP > 70 mm Hg (%GMT)	− 0.21^b^	− 0.08
CPPopt		
∆CPPopt < − 5 mm Hg (%GMT)	0.02	− 0.04
∆CPPopt ± 5 mm Hg (%GMT)	0.22^b^	0.20^c^
∆CPPopt > 5 mm Hg (%GMT)	− 0.12	− 0.08

The %GMT of each cerebral physiological variable and threshold was analyzed in relation to GOS-E for each group (significant contusions and small/no contusions). The Spearman’s rank correlation coefficient is presented

CPP, cerebral perfusion pressure, CPPopt, optimal CPP, GMT, good monitoring time, GOS-E, Glasgow Outcome Scale-Extended, ICP, intracranial pressure, IQR, interquartile range, PRx, pressure reactivity index

^a^Statistical significance at *p* < 0.001

^b^Statistical significance at *p* < 0.05

^c^Statistical significance at *p* < 0.01

In the cohort with small/no contusions, a higher %GMT of PRx > 0.30, a lower %GMT of CPP within 60–70 mm Hg, and a lower %GMT of ∆CPPopt ± 5 mm Hg were associated with lower GOS-E scores (Table 4). Excluding those patients who had undergone DC from the analyses between PRx/∆CPPopt and GOS-E did not alter the associations. In a heatmap with the %GMT of PRx/CPP combinations (Fig. 1c), there was a transition toward unfavorable outcome when CPP exceeded 80 mm Hg, which was more pronounced when PRx exceeded approximately 0.25. In similar analyses of PRx/ΔCPPopt in relation to GOS-E (Fig. 2c), there was a transition toward unfavorable outcome for both negative and positive ΔCPPopt when PRx was above approximately 0.25.

### Multiple Logistic Outcome Regressions of Unfavorable Outcome

In multiple logistic outcome regressions of unfavorable outcome (Table 5), age, GCS M, unreactive pupil(s), and the %GMTs of ICP > 20 mm Hg, PRx > 0.30, and CPP within 60 to 70 mm Hg were used as independent variables. In such a regression, based on the significant contusion cohort, a lower %GMT of CPP within 60–70 mm Hg was the only physiological variable independently associated with a higher rate of unfavorable outcome. In the small/no contusions cohort, a higher %GMT of PRx > 0.30 was the only physiological variable independently associated with a higher rate of unfavorable outcome. In similar regressions, in which the %GMT of CPP within 60–70 mm Hg was replaced with a %GMT of ΔCPPopt ± 5 mm Hg, no physiological variable turned out significant in the significant contusions cohort. In a similar regression with the small/no contusions cohort, a higher %GMT of PRx > 0.30 and a lower %GMT of ΔCPPopt ± 5 mm Hg were independently associated with a higher rate of unfavorable outcome. Older age, lower GCS M score, and unreactive pupil(s) were also associated with a higher rate of unfavorable outcome.

**Table 5:** Multiple logistic outcome regressions of unfavorable outcome

Variables	Significant contusions, OR (95% CI)		Small/no contusions, OR (95% CI)	
	Absolute CPP^a^	CPPopt^b^	Absolute CPP^c^	CPPopt^d^
Age (years)	1.04 (1.01–1.07)^e^	1.04 (1.01–1.07)^f^	1.03 (1.01–1.05)^g^	1.03 (1.01–1.04)^g^
GCS M (scale)	0.42 (0.22–0.76)^f^	0.44 (0.22–0.78)^f^	0.68 (0.52–0.87)^g^	0.66 (0.50–0.85)^f^
Unreactive pupil(s) (yes)	2.51 (0.64–11.53)	2.08 (0.56–8.98)	1.83 (0.90–3.74)	2.27 (1.07–4.88)^e^
ICP > 20 mm Hg (%GMT)	1.02 (0.99–1.05)	1.00 (0.98–1.03)	1.02 (0.99–1.04)	1.00 (0.98–1.03)
PRx > 0.3 (%GMT)	0.99 (0.95–1.02)	0.99 (0.96–1.03)	1.03 (1.01–1.05)^e^	1.03 (1.01–1.05)^f^
CPP 60 to 70 mm Hg (%GMT)	0.96 (0.92–0.99)^e^	NA	0.99 (0.97–1.01)	NA
∆CPPopt ± 5 mm Hg (%GMT)	NA	0.95 (0.89–1.01)	NA	0.96 (0.92–1.00)^e^

AIC, Akaike information criterion, AUROC, area under receiver operating characteristic curve, CI, confidence interval, CPP, cerebral perfusion pressure, CPPopt, optimal CPP, GCS M, Glasgow Coma Scale motor score, GMT, good monitoring time, ICP, intracranial pressure, NA, not applicable, OR, odds ratio, PRx, pressure reactivity index

^a^AIC = 147, AUROC (95% CI) = 0.78 (0.70–0.87), Nagelkerke = 0.31

^b^AIC = 149, AUROC (95% CI) = 0.76 (0.67–0.84), Nagelkerke = 0.28

^c^AIC = 314, AUROC (95% CI) = 0.78 (0.72–0.83), Nagelkerke = 0.28

^d^AIC = 304, AUROC (95% CI) = 0.78 (0.73–0.84), Nagelkerke = 0.29

^e^Statistical significance at *p* < 0.05

^f^Statistical significance at *p* < 0.01

^g^Statistical significance at *p* < 0.001

## Discussion

In this study, the main findings were that patients with TBI with significant cerebral contusions recovered more favorably if CPP was below 70–80 mm Hg, and even values below 60 mm Hg could overall be tolerated. However, elevated PRx and ΔCPPopt were only weakly associated with outcome in this TBI subtype. On the contrary, in patients without significant contusions (i.e., those with diffuse injury, focal extra-axial hemorrhages, or smaller contusions), PRx and ΔCPPopt were independently associated with outcome. Our findings suggest that PRx and CPPopt, which reflect the global cerebral pressure autoregulation, may be most relevant in patients without large contusions but less valid in patients with predominant focal brain lesions. In the latter group, focus should rather be to avoid high absolute CPP levels. However, these findings are hypothesis generating and need to be validated in prospective trials before implementation into clinical practice.

The association between the cerebral physiological variables and outcome differed between patients with TBI with significant contusions and those with small/no contusions. In the TBI cohort with significant contusions, there was a clear transition toward unfavorable outcome when CPP exceeded 80 mm Hg, as illustrated in the heatmaps. This finding may reflect that these patients were particularly vulnerable to develop detrimental brain edema with high CPP [26], whereas CPP slightly below 60 mm Hg could usually be tolerated [35]. It was clear in these heatmaps that the association between CPP and GOS-E did not change depending on the concurrent PRx. This fact could be explained by the fact that PRx is a global measure of cerebral pressure autoregulation and, thus, may not be sensitive to the regional autoregulatory and blood flow disturbances near the focal lesion [36]. Although PRx and ΔCPPopt were not independently associated with clinical outcome in the multiple logistic regressions, there was a trend toward unfavorable outcome in the PRx/ΔCPPopt plot for these patients when ΔCPPopt was above 10 mm Hg in combination with PRx above 0.25. This combination of PRx/ΔCPPopt likely reflected when the upper limit of autoregulation was exceeded and hyperemia took place in the brain [37]. This finding suggests that the interpretation of PRx/ΔCPPopt combinations still may be of some clinical value in this TBI subtype. Otherwise, a higher %GMT of ΔCPPopt ± 5 mm Hg was only marginally associated with better outcome and did not hold as an independent variable in the multiple regressions in these patients. Altogether, our results indicate that patients with large cerebral contusions exhibit a significant focal injury and that global metrics of the cerebral autoregulatory status, including PRx and CPPopt, may then be less valid. Instead, in this particular subgroup, it seems that elevated CPP should be avoided because it may worsen brain edema. These ideas are consistent with the Lund concept [4]. Although the role of individualized therapy based on global cerebral autoregulatory variables appears less promising in these patients, it is possible that CPP management could still be further individualized by other means, such as focal neuromonitoring of brain oxygenation and energy metabolism (microdialysis) [2]. These methods could aid in determining when hypoxia and energy metabolic decompensation start to occur in the contusional penumbra because of low CPP with focal ischemia [2, 35].

For patients with small/no significant contusions, elevated PRx and CPP deviation from CPPopt were independently associated with a lower rate of favorable outcome. In this group, CPP within 60–70 mm Hg was also associated with favorable outcome, but it was weaker and did not hold true in multiple regression analysis. Thus, the role of global autoregulatory metrics may be of greater validity to further individualize neurointensive care management in this TBI subtype. However, as stated previously, this needs to be corroborated in prospective trials.

A strength of this article was the large patient cohort with available high-frequency physiological monitoring data. We also used a novel approach, developed by our group [17], to study combinations of cerebral perfusion variables, including PRx/CPP and PRx/ΔCPPopt, in relation to outcome.

There were also some limitations. This was a single-center study, and our findings are reflections of the cohort demographics, brain injury patterns, and management, which may limit the external validity. The study design was observational and exploratory. The associations found in the results may not be causal but could reflect potential confounding variables, although this was taken into account to some extent in the multiple regressions. Furthermore, it has been questioned if PRx and CPPopt are reliable in the case of an open EVD or post-DC. Using an EVD system with a certain outflow resistance preserves much of the ICP amplitude when the EVD is open and makes the measurements sensitive for very rapid ICP changes, which is a prerequisite for reliable PRx calculations that were based on 10-s averages of high-resolution data. Several studies support that these measures remain valid in these scenarios [38–40], and we therefore decided not to exclude these patients. In addition, the EVD was typically closed or only intermittently opened in the majority of patients with this monitoring type. The cutoff for significant contusions at 10 mL was chosen to capture a sufficiently large focal lesion that could have a clinical impact without necessarily requiring surgery. The cutoff was chosen a priori and was similar to the one used in the STITCH trial [31]. Ten milliliters was also deemed as the lower end of what was considered a significant contusion in a recent Collaborative European NeuroTrauma Effectiveness Research in Traumatic Brain Injury (CENTER-TBI) study [41]. We considered studying patients with isolated contusions without other concurrent lesions, e.g., aSDH and tSAH, but this would both limit the size of the patient cohort in this study and reduce the external validity of our findings to a minority of all patients with TBI. Although the patients in the significant contusion group also had other lesions, these were typically small. However, in those cases when the concurrent extra-axial lesions were significantly large, they were often evacuated, and the patients then still had their significant contusion injury with leaky vessels, which likely impacted their clinical course. Furthermore, some of the large contusions were evacuated, and these patients were kept in the significant contusions group because the postoperative intracerebral cavity and the pericontusional area were still expected to exhibit leaky vessels. Thus, for all of these reasons, we consider our simple contusion dichotomization justified. In addition, the associations between cerebral physiology and outcome were weak; however, this was expected, taking into account the multitude of factors (e.g., demographic factors, primary brain injury, secondary brain injuries, and rehabilitation) that influence long-term functional outcome [42]. Also, the analyses focused on the most acute phase (first week), although secondary injuries (e.g., due to edema evolution) occasionally occur later. Furthermore, the radiological assessments were performed by only one of the authors (TSW), which potentially may have decreased the accuracy, reliability, and interpretation of the results. However, the volume calculations were automated using the Brainlab software, and the delineation of regions of interests was generally simple, which makes it unlikely that the number of inaccurate measurements would have affected the results significantly. Lastly, the odds ratios of the physiological variables in Table 5 overlapped between the significant contusions and the small/no contusions groups in many cases, which indicates that the differences between these cohorts were relatively small.

## Conclusions

In this study, the main findings were that patients with significant cerebral contusions recovered more favorably if CPP was within 60–70 mm Hg, whereas particularly higher values did not appear to be well tolerated. However, elevated PRx and CPP deviation from CPPopt were only weakly associated with outcome in this subgroup. On the contrary, in patients without significant contusions, PRx and ΔCPPopt were independently associated with outcome. Our findings suggest that PRx and CPPopt, which reflect the global cerebral pressure autoregulation, may be of less relevance in patients with predominant large cerebral contusions, whereas these global cerebral autoregulatory variables may be more useful in patients without significant focal intracerebral lesions. Instead, among the patients with TBI with significant contusions, higher CPP, above 80 mm Hg, could be particularly detrimental and should be avoided. However, this was an observational, hypothesis-generating study, and our findings need to be validated in prospective trials before implementation into clinical practice.

## Supplementary Information

Below is the link to the electronic supplementary material.

Supplementary file1 (DOCX 14 kb)
