# Supplementary material for: Should Patients with Traumatic Brain Injury with Significant Contusions be Treated with Different Neurointensive Care Targets?
Source: Neurocrit Care. 2024 Mar 20;41(2):511–22. doi: 10.1007/s12028-024-01954-y (PMC11377649; doi:10.1007/s12028-024-01954-y)
Supplement: Supplementary file 1 — Supplementary file1 (DOCX 14 kb) [file 12028_2024_1954_MOESM1_ESM.docx]

**Supplementary table 1. Hematoma evacuation in the significant contusion and small/no contusion cohort**

| **Type of evacuated hematoma** | **All** | **Significant ontusion** | **Small/no contusion** |
| --- | --- | --- | --- |
| EDH, n (%) | 24 (6%) | 2 (2%) | 22 (8%) |
| EDH + aSDH, n (%) | 6 (2%) | 3 (3%) | 3 (1%) |
| EDH + contusion, n (%) | 2 (1%) | 2 (2%) | 0 (0%) |
| aSDH, n (%) | 92 (24%) | 14 (12%) | 78 (29%) |
| aSDH + contusion, n (%) | 22 (6%) | 22 (18%) | 0 (0%) |
| IVH, n (%) | 1 (0.3%) | 0 (0%) | 1 (0.4%) |
| Contusion, n (%) | 32 (8%) | 32 (27%) | 0 (0%) |
| Total, n (%) | 179 (46%) | 75 (63%) | 104 (39%) |

The proportions were calculated for each cohort (all, significant contusion, and small/no contusion).

aSDH = Acute subdural hematoma. EDH = Epidural hematoma. IVH = Intraventricular hemorrhage.
